# Wild-type Yellow fever virus in cerebrospinal fluid from fatal cases in Brazil, 2018

**DOI:** 10.3389/fviro.2022.936191

**Published:** 2022-09-09

**Authors:** Izabela Mauricio de Rezende, Adriana Regina Campolina Cenachi, Thais Alkifeles Costa, Gabriela Fernanda Garcia Oliveira, Livia Rabelo, Leticia Mattos Menezes, Indiara Penido, Leonardo Soares Pereira, Matheus Soares Arruda, Andreza Parreiras Gonç alves, Pedro Augusto Alves, Erna Geessien Kroon, Carlos Eduardo Calzavara-Silva, Dario Brock Ramalho, Olindo Assis Martins-Filho, Andrea Teixeira-Carvalho, A. Desiree LaBeaud, Betânia Paiva Drumond

**Affiliations:** 1 Laboratory of Viruses, Microbiology Department, Biological Sciences Institute, Federal University of Minas Gerais, Minas Gerais, Brazil; 2 Department of Pediatrics, Division of Infectious Disease, Stanford University School of Medicine, Stanford, CA, United States; 3 Eduardo de Menezes Hospital, Belo Horizonte, Brazil; 4 Bendigo Heath Hospital, Bendigo, VIC, Australia; 5 Immunology of Viruses Diseases, René Rachou Institute, Oswaldo Cruz Foundation/FIOCRUZ, Minas Gerais, Brazil; 6 Cellular and Molecular Immunology, René Rachou Institute, Oswaldo Cruz Foundation/FIOCRUZ, Minas Gerais, Brazil; 7 Secretary of Health of Minas Gerais state, Belo Horizonte, Brazil; 8 Integrated Group of Biomarkers Research, René Rachou Institute, Oswaldo Cruz Foundation/FIOCRUZ, Minas Gerais, Brazil

**Keywords:** Yellow fever virus, yellow fever, cerebrospinal fluid, wild-type Yellow fever virus, neurotropic, central nervous system

## Abstract

Yellow fever virus (YFV) is the causative agent of yellow fever (YF), a hemorrhagic and viscerotropic acute disease. Severe YF has been described in approximately 15–25% of YF patients, with 20–50% of severe YF cases being fatal. Here we analyzed cerebrospinal fluid (CSF) samples collected during the YF outbreak in Brazil in 2018, aiming to investigate CNS neuroinvasion in fatal YFV cases. YFV RNA was screened by RT-qPCR targeting the 3’UTR region of the YFV genome in CSF. CSF samples were tested for the presence of anti-YFV IgM and neutralizing antibodies, coupled with routine laboratory examinations. Among the 13 patients studied, we detected anti-YFV IgM in CSF from eight patients and YFV RNA in CSF from five patients. YFV RNA genomic load in CSF samples ranged from 1.75×103 to 5.42×103 RNA copies/mL. We genotyped YFV from three CSF samples that grouped with other YFV samples from the 2018 outbreak in Brazil within the South-American I genotype. Even though descriptions of neurologic manifestations due to wild type YFV (WT-YFV) infection are rare, since the last YF outbreak in Brazil in 2017–2018, a few studies have demonstrated WT-YFV RNA in CSF samples from YF fatal cases. Serological tests indicated the presence of IgM and neutralizing antibodies against YFV in CSF samples from two patients. Although the presence of viral RNA, IgM and neutralizing antibodies in CSF samples could indicate neuroinvasiveness, further studies are needed to better elucidate the role of YFV neuroinvasion and possible impacts in disease pathogenesis.

## Introduction

Yellow fever virus (YFV) causes approximately 30,000 deaths out of 200,000 infections annually in tropical Africa and South America (1, 2). Severe YF has been described in approximately 15–25% of yellow fever (YF) patients, with 20–50% of severe YF cases being fatal (1, 3). YF is a viscerotropic hemorrhagic disease in humans, causing a variety of signs and symptoms including fever, headache, vomiting, jaundice, chills, nausea, abdominal pain, myalgia, arthralgia, rash, diarrhea, and bleeding, among others (1, 4). In patients with severe clinical manifestations, high levels of aspartate (AST) and alanine aminotransferases (ALT), thrombocytopenia, and a multifactorial bleeding disorder caused by reduced synthesis of clotting factors are usually observed. Acute liver disorders are considered one of the main factors causing brain pathology. Indeed, cytokine storm developed during the YF viscerotropic disease could contribute to central nervous system inflammation and YFV neuroinvasion (5).

The currently attenuated vaccine strains derived from the lineage 17D were originated from wild-type virus strain Asibi (6). This vaccine can cause adverse events such as YF vaccine-associated viscerotropic disease (YEL-AVD), which resembles the disease caused by WT-YFV, and YF vaccine-associated neurotropic disease (YEL-AND) with infection of the central nervous system (CNS). YEL-AND includes at least one of the following signs and symptoms: fever higher than 38° C, headache, focal neurological symptom (including ataxia, aphasia, and paresis), meningeal signs, altered mental status, seizure and cerebrospinal fluid (CSF) pleocytosis (7). Both YEL-AVD and YEL-AND are rare but often fatal (6). It is believed that these severe adverse events are influenced mainly by host factors, including the immune response. In contrast, wild-type YF (WT-YYF) infection in brain tissue, causing viral encephalitis, is rarely described (8).

Despite the viscerotropic aspect of YFV infection, since the last YF outbreak in Brazil in 2016–2019, few studies have detected WT-YFV genomic RNA in CSF from fatal cases (3, 9, 10). CNS involvement has been associated with other flavivirus infections such as Japanese encephalitis virus, West Nile virus, Zika virus (ZIKV), and dengue virus (DENV) (9, 11–16). The clinicopathological presentations of flavivirus CNS infection range from mild meningitis to fulminant meningoencephalomyelitis (16). These viruses usually reach the CNS through blood and brain-blood barrier (BBB) disruption (16, 17) due to increased endothelial cell permeability induced by viral replication or neuroinflammation (18). More recently, a new extension of the CNS was found, described as a network of lymphatic vessels within the dura mater that runs alongside blood vessels. This network provides an alternate conduit for the drainage of immune cells and CSF from the brain (19), and could also be involved during CNS invasion. This data suggests that BBB permeability and CNS lymph drainage in the setting of an exaggerated proinflammatory cytokine response may lead to the shock phase of yellow fever (1). The goal of this study was to further investigate CNS neuroinvasion in fatal YFV cases during the YF outbreak in Brazil in 2018.

## Methodology

### Biological samples and ethics

All patients were admitted at Eduardo de Menezes Hospital (HEM), Belo Horizonte, Brazil, a reference hospital for infectious diseases in Minas Gerais state, during the 2018 YF outbreak. Diagnosis of YF was confirmed through positive YFV RT-qPCR, YFV isolation from serum sample, or detection of anti-YFV IgM in serum, followed by negative anti-DENV and anti-ZIKV IgM tests. Diagnostic tests were run at Reference Laboratory in Minas Gerais (Ezequiel Dias Foundation), following routine tests at this institution. Test results were referred to us. Of the 53 fatal cases admitted at HEM during the 2018 YF outbreak, 13 underwent postmortem CSF collection. CSF was collected at the Eduardo de Menezes Hospital and kept in liquid nitrogen until adequate transport to the Laboratory of Viruses/UFMG/Brazil and stored at −70 °C. Ethics Committee on Human Research approved the research at René Rachou Institute/FIOCRUZ on CAAE 65814417.0.0000.5091 and CAAE: 43000815.7.0000.5091 and at Stanford University School of Medicine, under the eProtocol number 53676.

### YFV investigation

Total RNA extraction was done using 140 μL of CSF sample and QIAmp Viral RNA Mini kit (Qiagen, Germany), following the manufacturer’s instructions. Total RNA (5 μL) was used in RT-qPCR targeting the 3’UTR region of the YFV genome (20). Positive samples were then used for quantitative PCR, using Bio Gene Research Yellow Fever PCR kit, following manufacturer’s instructions (Bioclin, Brazil).

### YFV genotyping

YFV RNA isolated from CSF samples were subjected to genotyping, as previously described (21). Briefly, part of NS5 gene was amplified (260 bp) (22) and sequenced (23). The sequences generated here were then aligned with a sequence panel previously used (21), using Clustal W, implemented on Mega7 (24). The Maximum-likelihood tree was generated using Kimura-2-parameters nucleotide substitution model with gamma distribution, and 1,000 bootstraps replicates, using MEGA7 (24).

### Serological analysis

An anti-YFV IgM immunochromatographic test was run (ECO Diagnóstica, Brazil) on CSF samples. YFV RNA positive CSF samples that were also IgM anti-YFV reactive were used in plaque reduction neutralization test (PRNT), as previously described (25). Briefly, CSF samples were two-fold diluted from 1:20 to 1:1280. Dilutions of CSF were incubated with 150 plaque-forming units (PFUs) of YFV vaccine strain YFV-17DD. The mix of CSF and vaccine YFV-17DD were then used for Vero cells’ infection. After five days post-infection, cells were fixed, stained using crystal violet, and viral plaques were counted. Samples were considered positive when presented with a reduction of 50% of total counted plaques compared to virus control. Samples were tested in duplicate in PRNT.

### Laboratory exams

As part of the Intensive Care Unit (ICU) routine of HEM, a complete hemogram was run, including counting of hemoglobin, hematocrit, platelets, neutrophils, leukocytes, lymphocytes, eosinophils. Routine tests also included blood urea, albumin, AST, ALT, gamma-glutamyl transferase (GGT), alkaline phosphatase (ALP), lactate, total (Tbil) and direct bilirubin (Dbil), creatinine (Cr), and International normalized ratio (INR). Values for each exam were following the Laboratory Test Ranges (26).

### Statistical analysis

Considering the small number of patients in this study, we performed the nonparametric Mann-Whitney U test comparing laboratory variables regarding patients with and without YFV RNA detection in CSF. Analyzed variables were selected according to previous studies that have been shown predictive factors for severe YF disease (3), and hepatic injury markers, including neutrophil count, ALT, AST, INR, indirect bilirubin, and creatinine on day of admission. Significative difference was considered when p < 0.05.

## Results

During the YF outbreak in Brazil in 2018, CSF samples were collected postmortem from 13 patients at HEM. Our results showed that, among those, five patients were RT-qPCR positive for YFV RNA in CSF. We estimated the YFV RNA genomic load in CSF samples from four patients, and values ranged from 1.75 × 103 to 5.42 × 103 RNA copies/mL (Table 1). Positive CSF samples were collected on the day of death, corresponding to 4 to 25 days post-symptoms (DPS) (Table 1).

The five YFV positive CSF samples were from male patients, ranging from 24 to 62 years of age (Table 2). At hospital admission, the most common clinical findings included fever, jaundice (5/5 patients), followed by headache, vomiting (4/5 patients); confusion, oliguria, myalgia (3/5 patients); asthenia, bleeding (2/5 patients); seizure, abdominal pain, diarrhea, dehydration, and palpable liver (1/5 patients) (Table 2). One patient died on the day of hospitalization (P #3) and four remained hospitalized from 4 up to 25 DPS (Table 2), until the day of death. Patients P #1, P #2, and P #5 had CNS signs at presentation (altered mental status). Patients #3 and #4 arrived at HEM already intubated, with a report of seizure and altered mental status.

Among the 13 CSF samples, 12 were tested using an IgM anti-YFV immunochromatographic test, and eight were IgM positive (Table 2). We could not run this test using CSF sample from P #13, due to insufficient quantity. Samples that had YFV RNA detected in CSF and were IgM reactive (P #1, P #2, P #4), were used in a PRNT assay. CSF samples from patients P #1 and P #4 presented neutralizing antibodies against YF 17DD strain (P #1: up to sera dilution 1:40 and P #4: up to sera dilution 1:640) (Table 1).

**FIGURE 1:**
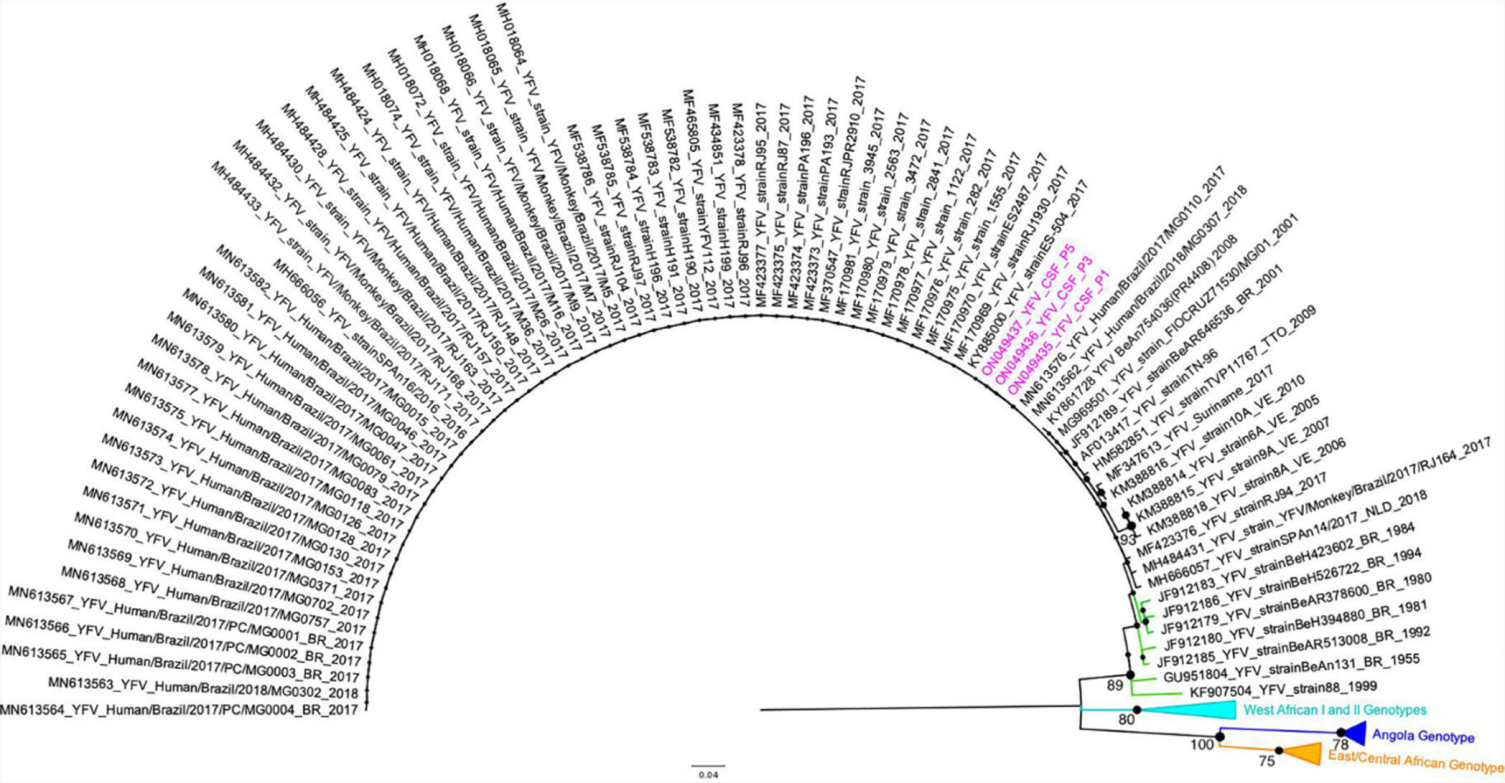
Maximum Likelihood tree of Yellow fever virus. The maximum clade credibility tree inferred using Yellow fever virus (YFV) sequences (187 nt) is shown (corresponding to position 9046 to 9232 compared to the YFV nucleotide sequence, GenBank accession number KY885001). The bootstrap values (1.000 replicates) are represented by circles drawn in scale in the nodes. Bootstrap values higher than 75 are indicated. Sequences generated in this study are highlighted in pink. The clade containing samples from the genotype South-American I is represented in black. The clade containing samples from genotype South-American II is represented in green. For clarity purposes, some branches representing different genotypes were collapsed and colored as follows: West African I and II (light blue), Angola (dark blue), and East/Central African (orange), respectively. The tree was reconstructed using the nucleotide substitution model kimura 2-parameters with 4-categories gamma distribution. The analysis was performed using MEGA7 and the tree was visualized and edited in FigTree v1.4.4.

Regardless the fact that none of the patients had been vaccinated against YF up to 30 days before symptoms onset, we performed YFV genotyping using CSF from three patients that presented YFV RNA (P #1, P #3, and P #5). The other two patients were negative after the genotyping RT-qPCR. All YFV RNA sequences detected in CSF were assigned to the South-American I genotype cluster, together with other YFV samples from the 2018 outbreak in Brazil (Figure 1).

Severe yellow fever cases could be associated with encephalopathy and altered liver injury markers, which can lead to an increase in the BBB permeability, facilitating the virus from crossing this barrier and being found in the central nervous system (16, 17). Considering previously described risk factors for severe YF (3) (older age, male sex, higher neutrophil counts, higher levels of AST, indirect bilirubin, creatinine, prolonged prothrombin time, and YFV RNA viral load in plasma) and hepatic injury markers, we performed statistical analysis comparing data from the five patients with positive CSF and the other eight patients with no detection of YFV RNA in CSF. None of the analyzed variables presented a statistically significant difference. However, all 13 patients presented with at least two risk factors for severe disease (Table 2).

## Discussion

This study demonstrates the presence of wild type YFV RNA in CSF from five naturally infected YF patients during the 2018 YF outbreak in Brazil. We were also able to detect antibodies against YFV in CSF in three of those five patients. These results suggest that YFV can be neuroinvasive in severe disease, but more studies are needed to confirm the role of YFV in neuroinvasion given our small sample size. Although YFV genomic RNA has also been detected in CSF of naturally infected patients by other studies (3, 9, 10), encephalitis caused by YFV neuroinvasion is rarely described (1, 8) and more studies are needed to better understand this phenomenon in humans. While neurological disease can also occur as an adverse event following YF vaccination, neurologic manifestations due to WT-YFV infection are rare, and early studies described delirium, seizures (1, 8, 27), and coma as one of the events preceding death due to YF infection (1). It is known that these manifestations are more associated with cerebral edema and metabolic factors associated with severe disease than with the action of the virus itself in the CNS (8).

According to protocols of patient management recommended by the Ministry of Health in Brazil, all 13 patients analyzed in this study were classified as YF severe cases (group C) and hospitalized in an intensive care unit (ICU) (7). This classification includes the presence of signs and symptoms as oliguria, somnolence, lethargy, mental confusion, coma, seizure, bleeding, difficulty to breathing, hypotension, poor perfusion or AST or ALT ≥ 2,000 UI/L, CR ≥2, INR ≥ 1,5, and platelets < 50,000 cells/mL (7). In addition, Kallas and colleagues (2019) described older age (> 45 years old), male sex, higher neutrophil counts (> 4,000 cells/mL), higher levels of AST (≥ 3,500 UI/L), indirect bilirubin (≥ 0.64 mg/dL), creatinine (≥2.36 UI/L), prolonged prothrombin time (≥1.46), and YFV RNA viral load in plasma (≥ 5.1 log copies/mL) as risk factors for fatal cases (3).

Analyzing our patients on the day of their death, they presented with indirect bilirubin, INR, and creatinine above the cut-off found by Kallas etal. (3), and all were considered comatose on the day of death. Four patients showed AST and neutrophil counts higher than cut-off values described as risk factors for fatal disease (3). In addition, two patients studied here presented with serum YFV genomic RNA load higher than the established threshold for fatal YF (3). All patients presented with three or more signs and symptoms described by the Ministry of Health (7) as an important factor for severe YF clinical illness (Table 2), and at least two several markers for poor prognosis, described by Kallas etal. (3) (3, 7), that could be lead to neurologic manifestations (8). However, we did not find any risk factor that could be associated with detection of YFV RNA in CSF.

Together, our findings suggest severe cellular damage by YFV in the five patients with YFV RNA in CSF. The combination of liver, kidney, and bone marrow damage indicates the involvement of multiple organs, resulting in a systemic damage. In most cases, this damage might be irreversible due to systemic impairment and the interdependence of organ systems in their functioning. Liver failure probably contributes to encephalitis (28), and the already fragile BBB allowed virus crossing to CNS, likely mediated by cytokine storm produced during viscerotropic YF infection. It has been described for other flaviviruses, that this cytokine storm can alter the permeability of the endothelium through the disturbance of the tight junctions of BBB (reviewed by 29). Bearing in mind that WT- YFV must have a relative neurovirulence in humans, the virus probably reached the CNS due to the severe clinical illness of the studied patients during YF infection. The virus could have reached the CNS as a free particle or inside an infected cell (Trojan-horse entry mechanism) (30). Possibly as a consequence of immune response against YFV, infected cells could have crossed the BBB carrying viruses’ particles and immunoglobins to the CNS (reviewed by 30). Another pathway that might have allowed neuroinvasion by YFV could be the recently described network of lymphatic vessels within the dura mater that runs alongside blood vessels and is responsible for fluid flow and lymphatic drainage from the central nervous system (19).

While the presence of IgM in CSF without direct detection of virus could indicate neuroinvasiveness, it is important to note that this phenomenon might be a consequence of a passive crossing through the BBB and does not necessarily implicate the presence of YFV particle or active virus replication in CNS. However, the recent YF outbreak in Brazil was caused by a new viral lineage with unique mutations and amino acid substitutions (2, 4) and the possible impacts of these alterations in viral biology and pathogenesis still need to be addressed. Nevertheless, it is plausible to consider that the presence of YFV RNA, and possibly viral particles in CNS, could have harmed the outcome of these patients.

## Conclusion

In this study YFV genomic RNA, IgM, and neutralizing antibodies were detected in CSF in fatal YF cases at HEM with presenting signs and symptoms of severe viscerotropic YF disease. Severe clinical illness may facilitate the virus invasion in CNS. Further studies are needed to better elucidate the role of CNS involvement in YF disease.

## Figures and Tables

**TABLE 1:** Laboratory results of patients with detection of YFV RNA in CSF.

ID	P #1	P #2	P #3	P #4	P #5

AGE	24	54	62	45	41
DPS – CSF sample	9	25	4	6	9
YFV RT-qPCR (CSF)	positive	positive	positive	positive	positive
Genomic viral load	3.85E+03	n/a	1.75E+03	4.47E+03	5.42E+03
IgM (CSF)	Reactive	NR	NR	Reactive	NR
PRNT_50_ (CSF)	1:40	n/a	n/a	1:640	n/a

P, Patient; DPS, days post symptoms; CSF, cerebrospinal fluid; YFV, Yellow fever virus; NR, non-reactive; n/a, not available; PRNT, plaque reduction neutralization test.

**TABLE 2:** Clinical data at admission and death day of 13 YF patients analyzed in this study.

GENERAL DATA	ID	P 1	P 2	P 3	P 4	P 5	P 6	P 7	P 8	P 9	P 10	P 11	P 12	P 13
	AGE	24	54	62	45	41	80	55	55	64	27	46	56	68
	SEX	male	male	male	male	male	male	male	male	male	male	male	male	male
	anti-YFV IgM (CSF)	reactive	reactive	NR	reactive	NR	reactive	reactive	reactive	reactive	NR	reactive	NR	n/a
	YFV VACCINE RECORD	2001	NV	NV	NV	NV	NV	NV	NV	NV	NV	NV	NV	NV

Admission day	DPS	6	13	4	1	5	3	5	11	2	3	3	3	2
Death day		9	25	*	6	9	7	*	41	8	4	9	6	7
Admission day	Fever	P	P	P	P	P	P	P	P	P	P	P	P	P
Death day		A	A	*	0	P	A	*	n/a	A	A	A	A	A
Admission day	Headache	P	P	A	P	P	A	P	P	P	P	A	P	P
Death day		0	0	*	A	A	A	*	n/a	A	A	A	A	A
Admission day	Confusion	P	P	A	0	P	A	A	P	A	P	P	A	A
Death day		0	P	*	A	A	A	*	n/a	P	A	A	P	A
Admission day	Seizure	P	A	A	0	0	A	A	A	A	P	P	A	A
Death day		A	A	*	0	0	A	*	n/a	A	A	A	P	P
Admission day	Other neurological disorder	coma	A	A	A	A	A	A	A	A	A	A	A	A
Death day		coma	coma	*	coma	coma	A	*	n/a	A	coma	A	A	A
Admission day	Jaundice	P	P	P	P	P	P	P	P	A	P	P	P	P
Death day		P	P	*	A	P	A	*	n/a	P	A	P	A	A
Admission day	Platelets	34,000	87,000	86,000	55,000	162,000	62,000	55,000	61,000	96,000	10,200	40,000	46,000	81,000
Death day	(150,000 – 400,000 mm3)	75,000	67,000	*	59,000	89,000	57,000	*	67,000	109,000	67,000	47,000	70,000	57,000
Admission day	Neutrophils	7,458	7,544	4,536	1,200	13,600	4,712	3,016	1,872	1,098	3,844	3,936	5,472	n/a
Death day	(2,000 – 8,250 mm3)	n/a	n/a	*	n/a	n/a	n/a	*	n/a	n/a	n/a	n/a	n/a	n/a
Admission day	Creatinine	7.2	1	4.7	1	8.1	4.4	5.2	1.9	1.4	1.3	0.6	5.7	1.7
Death day	(0.7 – 1.30 mg/dL)	6.8	5.5	*	5.6	5.6	6.1	*	4.9	5.6	5.9	4.4	4.7	4.6
Admission day	Albumin	2.7	1.9	n/a	2.9	3	2.5	2.6	2.5	3.6	2.8	3.3	2.1	3.2
Death day	(3.4 – 4.5 g/dL)	2.2	2.4	*	2.8	n/a	2.5	*	2.4		2.4	3.2	2.1	3.4
Admission day	AST	14,060	231	410	7,491	17,000	6,674	8,369	7,630	4	5,475	7,679	19,000	13,437
Death day	(10 – 40 U/L)	4,019	574	*	14,928	4,943	4,807	*	120	7,269	15,098	5,141	4,728	9,228
Admission day	ALT	5,681	668	3,800	5,029	13,544	4,417	4,000	2,390	4,227	176	5,059	4,708	6,178
Death day	(10 – 40 U/L)	1,896	171	*	5,790	2,735	2,376	*	52	131	2,049	3,705	878	2,324
Admission day	TBil	7.2	21.7	5	2.9	4.9	13.6	6.8	9.9	1.7	5.6	4.8	6.3	2.8
Death day	(0.3 – 1.0 mg/dL)	7.4	n/a	*	13.3	6.9	18.1	*	29.4	14.5	5.6	16.7	4.7	10.1
Admission day	Dbil	6.7	19.7	2.2	2.2	3.2	13	6.5	8.6	1.3	5.6	3.7	5.9	2.3
Death day	(0.1 – 0.3 mg/dL)	6.4	29.9	*	12	3.1	17.4	*	26	13.9	3.8	14.2	4.3	8.6
Admission day	GGT	210	172	n/a	151	n/a	422	165	397	82	169	661	189	n/a
Death day	(9 – 50 U/L)	156	126	n/a	194	164	346	*	108	131	96	188	116	186
Admission day	Aphos	158	109	n/a	120	234	215	77	187	84	n/a	68	109	n/a
Death day	(30 – 120 U/L)	254	199	n/a	394	982	206	*	224	121	147	98	104	212
Admission day	INR	3.99	1.78	4.82	2.39	9.27	1.47	1.99	2.01	1.82	6	1.57	1.89	1.31
Death day		2.86	1.69	*	4.61	> 5.00	1.15	*	1.17	n/a	3.4	2.95	1.97	1.58

* data from admission and death day are the same. P 1 – P 13, patient 1 to 13 DPS, days post symptom onset; NR, non-reactive; n/a, not available; NV, non-vaccinated; P, presence; A, absence; AST, aspartate aminotransferase; ALT, alanine aminotransferase; Tbil, Total bilirubin; Dbil, Direct bilirubin; GGT, gamma-glutamyl transferase; APhos, alkaline phosphatase; INR, international normalized ratio. Laboratory test reference ranges followed ABIM (26).

## Data Availability

The datasets presented in this study can be found in online repositories. The names of the repository/repositories and accession number(s) can be found in the article/Supplementary Material.
